# Supplementary material for: The causal relationship between the human gut microbiota and pyogenic arthritis: a Mendelian randomization study
Source: Front Cell Infect Microbiol. 2024 Nov 26;14:1452480. doi: 10.3389/fcimb.2024.1452480 (PMC11629706; doi:10.3389/fcimb.2024.1452480)
Supplement: Supplementary file 1 [file DataSheet1.zip › mendelian test/ebi-a-GCST90027728.csv_leave_one_out.pdf]

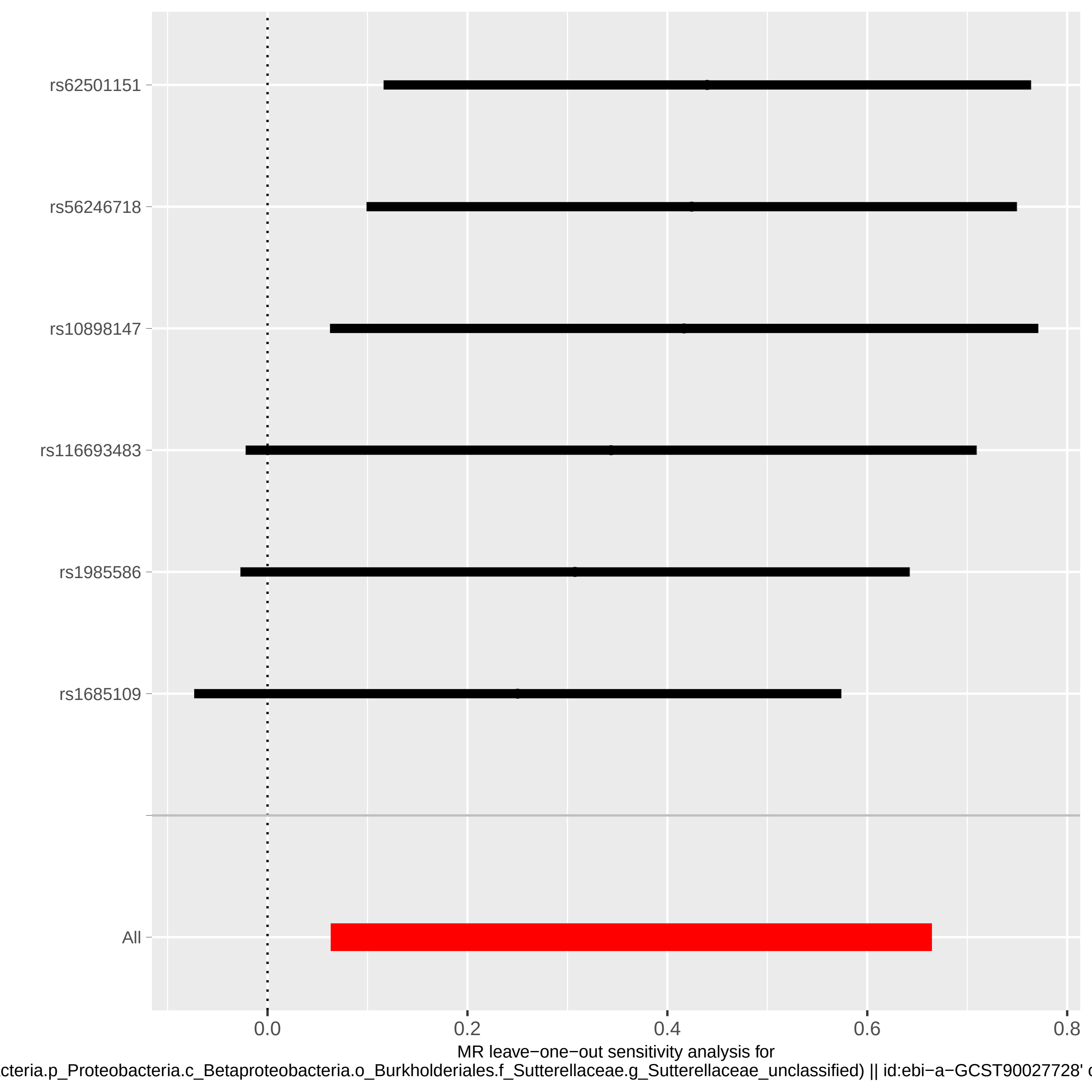
rs62501151
rs56246718
rs10898147
rs116693483
rs1985586
rs1685109
All
0.0
0.2
0.4
0.6
0.8
MR leave−one−out sensitivity analysis for
cteria.p_Proteobacteria.c_Betaproteobacteria.o_Burkholderiales.f_Sutterellaceae.g_Sutterellaceae_unclassified) || id:ebi−a−GCST90027728' o
